# Regional microbial signatures positively correlate with differential wine phenotypes: evidence for a microbial aspect to *terroir*

**DOI:** 10.1038/srep14233

**Published:** 2015-09-24

**Authors:** Sarah Knight, Steffen Klaere, Bruno Fedrizzi, Matthew R. Goddard

**Affiliations:** 1 School of Biological Sciences, University of Auckland, Private Bag 92019, Auckland Mail Centre, Auckland 1142, New Zealand; 2 Department of Statistics, University of Auckland, Private Bag 92019, Auckland Mail Centre, Auckland 1142, New Zealand; 3 School of Chemical Sciences, University of Auckland, Private Bag 92019, Auckland Mail Centre, Auckland 1142, New Zealand; 4 The School of Life Sciences, The University of Lincoln, Lincoln, LN6 7DL, United Kingdom

## Abstract

Many crops display differential geographic phenotypes and sensorial signatures, encapsulated by the concept of *terroir*. The drivers behind these differences remain elusive, and the potential contribution of microbes has been ignored until recently. Significant genetic differentiation between microbial communities and populations from different geographic locations has been demonstrated, but crucially it has not been shown whether this correlates with differential agricultural phenotypes or not. Using wine as a model system, we utilize the regionally genetically differentiated population of *Saccharomyces cerevisiae* in New Zealand and objectively demonstrate that these populations differentially affect wine phenotype, which is driven by a complex mix of chemicals. These findings reveal the importance of microbial populations for the regional identity of wine, and potentially extend to other important agricultural commodities. Moreover, this suggests that long-term implementation of methods maintaining differential biodiversity may have tangible economic imperatives as well as being desirable in terms of employing agricultural practices that increase responsible environmental stewardship.

Many important crops that comprise the same or very similar genotypes display differential geographic phenotypes in terms of the physical and sensorial signatures of their produce: this is generally encapsulated by the concept of *terroir*^1^. Often the chemical descriptors of these differential geographic phenotypes are well documented^2^,^3^,^4^,^5^,^6^,^7^,^8^,^9^; however, the factors that drive these differences remain elusive^10^. Classically, differential agricultural geographic phenotypes are thought to result from complex interactions between specific crop genotypes and local soils, topography, climate and agricultural practices, and these differential manifestations are commercially important as they add distinctiveness and thus value to products^10^. Microbes play key roles in the production of quality agricultural commodities for reasons ranging from their effect on crop nutrient availability via rhizosphere interactions with roots, through to their role in crop disease pressure: ultimately microbes influence plant and fruit health^11^,^12^,^13^. Additionally microbes transform plant products to economically and socially important commodities such as coffee, chocolate, bread, beer and a range of other fermented beverages including wine^14^. The potential contribution of, and link between, microbes and differential geographic phenotypes, or *terroir*, of agricultural products is assumed to exist, but to date has not been objectively verified^1^,^15^,^16^,^17^.

Wine has been made by humans since the dawn of civilization and is an important social and economic commodity. It arguably displays the strongest geographic signatures of all agricultural products and thus is a superb model to evaluate the degree to which there might be a microbial aspect to *terroir*. However, even for wine the drivers of *terroir* remain largely untested^10^. Microbes, predominantly fungi, may significantly affect the ‘phenotype’ of wine firstly by affecting grapevine and fruit health and development, and thus quality^18^, and secondly by manipulating wine flavor, aroma and style due to their actions during fermentation^19^,^20^. During alcoholic fermentation fungi including *Saccharomyces cerevisiae*, the primary yeast involved in wine fermentation, not only convert sugars into ethanol but also produce an array of secondary metabolites, including volatile compounds, that are important to wine aroma and flavor^21^,^22^. While grape-derived compounds may provide varietal distinctions, at least yeast-derived acids, alcohols, carbonyl compounds, phenols, esters, sulfur compounds and monoterpenoids all significantly contribute to wine quality and aroma^22^,^23^.

It is well documented that different species of microbes differentially affect vine health and development, and that different species of yeast, and even different genotypes of *S. cerevisiae*, produce different aroma profiles in wine^18^,^19^,^24^,^25^. Only recently has evidence been provided for the regional delineations of both microbial communities, and populations of *S. cerevisiae,* associated with vines and the populations driving the spontaneous ferment of fruit from these vines into wine^1^,^16^,^17^. On the face of it, together, these two sets of observations might seem enough to conclude that microbes have an influence on differential agricultural geographic signatures, at least for wine. However, the critical assumption here is that there is a positive correlation between microbial relatedness, and aroma profiles in wine: i.e. that closely related microbes and their communities produce closely related agricultural geographic signatures. This has not been shown, but here we provide the first evidence for such a link.

To evaluate this idea we focused on the potential for microbes to influence differential geographic wine phenotypes via fermentation. Wine may be made by either attempting to remove the array of microbes that are naturally associated with grapes and then deliberately inoculating with a commercial strain of yeast, or allowing the microbes naturally associated with grapes to conduct the ferment^26^. The former inoculated option reduces the potential for microbes to contribute to *terroir*, during fermentation at least, and has only been available commercially to winemakers since 1965^27^. The latter has been employed by humans since the dawn of civilization and is known as spontaneous or wild fermentation, and may comprise at least tens of species and hundreds of strains of *S. cerevisiae*^1^,^28^. Since spontaneously fermented wine comprises a diversity of yeast species and strains of *S. cerevisiae*, metabolic interactions between these different types may also potentially be the key to any microbial signature contributing to *terroir.* Due to the complex and often unpredictable nature of microbial interactions, community effects on the chemical and sensorial properties of wine are hard to experimentally control. As a first step towards understanding the impact microbes have on the regional distinctiveness of wine, we focus on the dominant species driving fermentation: *S. cerevisiae*.

We have recently shown there are genetically differentiated natural sub-populations of *S. cerevisiae* associated with vineyards and spontaneous ferments in major regions in New Zealand (NZ)^17^. Using population genetic analyses, here we select appropriate genetic representatives from these regional *S. cerevisiae* sub-populations and analyze their fermentative effects on a suite of chemicals known to significantly affect the phenotype of wine. We test for the presence of correlations between the genetic relatedness of these natural regional *S. cerevisiae* sub-populations and their resulting wine phenotypes, to conduct the first empirical test for whether there is a microbial aspect to *terroir*.

## Results

### Selection of *S. cerevisiae* genotypes

Recently Knight and Goddard^17^ isolated 3,900 *S. cerevisiae* from native forests, vineyards, and the spontaneous ferments of *Vitis vinifera* var Sauvignon Blanc fruit from six major regions in NZ (Hawke’s Bay, Martinborough, Nelson, Wairau Valley, Awatere Valley and Central Otago). Microsatellite genotype profiling of these isolates revealed the presence of 295 different genotypes. Bayesian population structure methods, and statistical analyses of the resulting ancestry profiles^29^, showed significantly distinct sub-populations residing in each of these regions^17^. Here we use the genetic ancestry profiles produced from Bayesian analysis, in combination with regional allele frequencies, to select *S. cerevisiae* genotypes from each of these regional sub-populations that span and represent the genetic diversity within each region. Genotypes that belong to the main inferred population correlating with each region, that also maximize the diversity of alleles present in each region, were selected, including at least one genotype that harbored regionally unique alleles. Supplementary Table S1 shows the Bayesian ancestry profiles for the genotypes originally analyzed by Knight and Goddard^17^: the inferred sub-populations common in each regions are noted and the genotypes selected for use in this study are highlighted. The allele frequencies within each regional population are shown in Supplementary Table S2 with the alleles harbored by the selected genotypes in this analysis highlighted. Due to the large diversity of alleles observed in each regional population, and the constraints on the number of ferments we could perform and analyze here, clearly not every allele could be represented. Rather, we included those genotypes harboring the more common alleles in each region: genotypes selected ensured that the average proportion of each population that harbored the represented alleles was no lower than 60% (Supplementary Table S2).

### Ferment Performance

All ferments were conducted using the same commercially derived batch of homogenized and sterilized Sauvignon Blanc juice from Marlborough in NZ. Six individual *S. cerevisiae* genotypes from each region, and co-inoculations of all six genotypes representing regional populations, were fermented in triplicate across three separate batches totaling 126 ferments. The extent to which sugars were fermented was analyzed by weight loss^30^, and most lost approximately 25 g indicating complete fermentation given the 220 g of sugar in the juice initially. One genotype from the Wairau Valley failed to ferment at all and was removed from all analyses. Eleven single genotype ferments, all in the third batch, displayed significantly less weight loss than the remaining ferments (*F*_1, 108_ = 905.9, *P *< 0.0001), indicating incomplete fermentation which is known to affect the volatile profiles of wines^31^. Also consistent with incomplete fermentation, the final concentration of ethanol in these ferments reduced (Dataset S1). It would also be expected that these ferments would have higher residual sugar but curiously the residual sugar reported for all of these ferments is below 2.5 g/L suggesting the majority of the sugar has been consumed (Dataset S1). To confirm this observation the wines from the third batch were also analyzed for residual sugar using an alternate enzymatic assay (Megazyme D-Fructose/D-Glucose assay kit), which confirmed the low residual sugar levels, reporting concentrations between 0–1.1 g/L. This suggests these ferments may not have had as much sugar at the start of fermentation, potentially caused by incomplete mixing of the initial juice before allocation into flasks. We therefore conservatively removed these ferments from all further analyses. Lag phase, the time taken for fermentation to initiate, differed significantly between batches (*F*_2, 89_ = 7.73, *P* = 0.0008), and since each batch contained one replicate of each sample, this was controlled for in subsequent statistical analyses by introducing a “batch” factor.

### Chemical profiles produced by single genotype ferments correlate with region of microbe origin

We quantified the concentrations of 39 volatile compounds and wine quality parameters produced in each of the 112 successful ferments using targeted GC-MS and FTIR analyses. First we analyzed the volatile profiles deriving from ferments conducted by single yeast genotypes only. A Permutational Multivariate Analysis of Variance (PERMANOVA) employing a full factorial model with “region” and “batch” as main effects, and where permutations kept replicates of each genotype together, revealed that both factors significantly affected volatile profiles (both *P* = 0.001), but provided no evidence of an interaction between these main effects (Table 1a). The R^2^ value for the region effect was greatest reporting the geographic origin of the *S. cerevisiae* genotypes explained approximately 10 % of the total variation in the chemical profiles (Table 1a). The lack of significance for the interaction term indicates this result is not confounded by the differences between batches. In addition, we analyzed these differential chemical profiles by accounting for human perception thresholds of compounds. Where available, we used empirically determined odor activity values (OAVs) to standardize the various chemical concentrations in these ferments^32^,^33^. The results of the subsequent PERMANOVA agreed with the initial analyses and again revealed a highly significant effect of the region of *S. cerevisiae* isolation on these wine phenotypes (Region: R^2^ = 0.127, *P* = 0.002; Table 1b). Thus, we can categorically reject the null hypothesis, and move to accept that there is a significant correlation between the region of isolation of *S. cerevisiae* and aroma profiles in wine.

**Figure 1:**
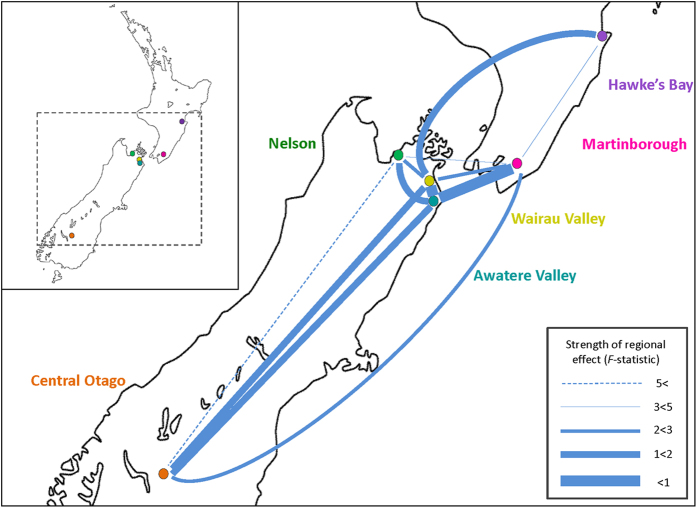
A map of the regions the tested genotypes of *S. cerevisiae* were isolated from and the strength of regional differentiation in the chemical profiles as indicated by *F*-statistics from pairwise PERMANOVA analyses (Supplementary Table S3). Wider lines indicate weaker regional distinctions in the chemical profiles produced (i.e. less distinct chemical profiles), while thinner lines indicate stronger regional distinction (i.e. more distinct chemical profiles). The inset indicates the portion of NZ highlighted in the larger map. The outline of the map of NZ was obtained from www.spraypaintstencils.com, where it is freely available, and all modifications were performed by the Authors in Microsoft Power Point.

Regional pairwise PERMANOVA analyses revealed different degrees of distinction between the chemical profiles produced by *S. cerevisiae* genotypes originating from different regions (Supplementary Table S3). *P*-values can be misleading when multiple comparisons are performed^34^, and it has been argued that more emphasis should be placed on the magnitude of the effect when dissecting differences^35^: we therefore examined the magnitude of the *F*-statistics from these multiple comparisons as a measure of the strength of evidence for a regional effect (i.e. the higher the *F*-statistic, the stronger the support for a regional effect). The chemical profiles of yeasts originating from Nelson are the most distinct compared to other regions with the mean of the pairwise *F*-statistics involving this region being the highest at 3.20 (Fig. 1; Supplementary Table S3). Nelson’s similarity to all regions is low with the exception of the Awatere Valley (Fig. 1). The Awatere and Wairau Valleys are the most similar to other regions (Fig. 1) and report the least distinct chemical profiles compared to other regions with mean *F*-statistics of 1.19 and 1.73 respectively (Supplementary Table S3). Central Otago, Martinborough and Hawke’s Bay are intermediate with a mix of both highly similar and more distinct relationships with other regions (Fig. 1; Supplementary Table S3).

**Figure 2:**
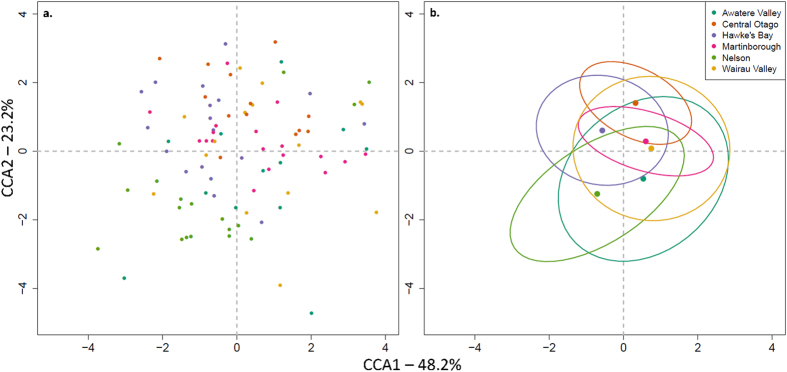
CCA of the 105 single genotype ferments analyzed. (**a**) All sample points colored by region. (**b**) Regional averages and 50 % ellipses.

To effectively visualize the differences in chemical profiles, the data were transformed and plotted using Constrained Correspondence Analysis (CCA)^36^. Overall a large overlap is observed between chemical profiles derived from genotypes from different regions (Fig. 2); however, the chemical profiles of Central Otago genotypes cluster in the upper half and those from Nelson mostly toward the lower left quadrant, with the exception of the three replicate samples from one genotype that are located in the upper right quadrant (Fig. 2a). The genotypes from Wairau and Awatere Valleys have the largest ellipses indicating a larger variability in the chemical profiles of these samples (Fig. 2b).

### Chemical drivers of regional differentiation in single ferment samples

Next we evaluated which components of the volatile profiles might be driving these differences in wine phenotype. Individual ANOVA analyses were performed for each of the chemical properties measured. As explained above, *F*-statistics are reported here in place of *P*-values as they are a more appropriate measure of support for multiple comparisons. We designate *F*-statistics larger than two as having a sizeable effect (i.e. region explains more the twice the variation in the model compared to the residuals), and thus 29 of the 39 compounds vary with respect to the region of origin of the yeast genotype (Supplementary Table S4). R^2^ values range from zero to 38% of the variation being explained by the *S. cerevisiae* genotype region of isolation, but no one class of chemical compound is exclusively responsible for the regional signal for wine phenotypes (Supplementary Table S4).

**Figure 3:**
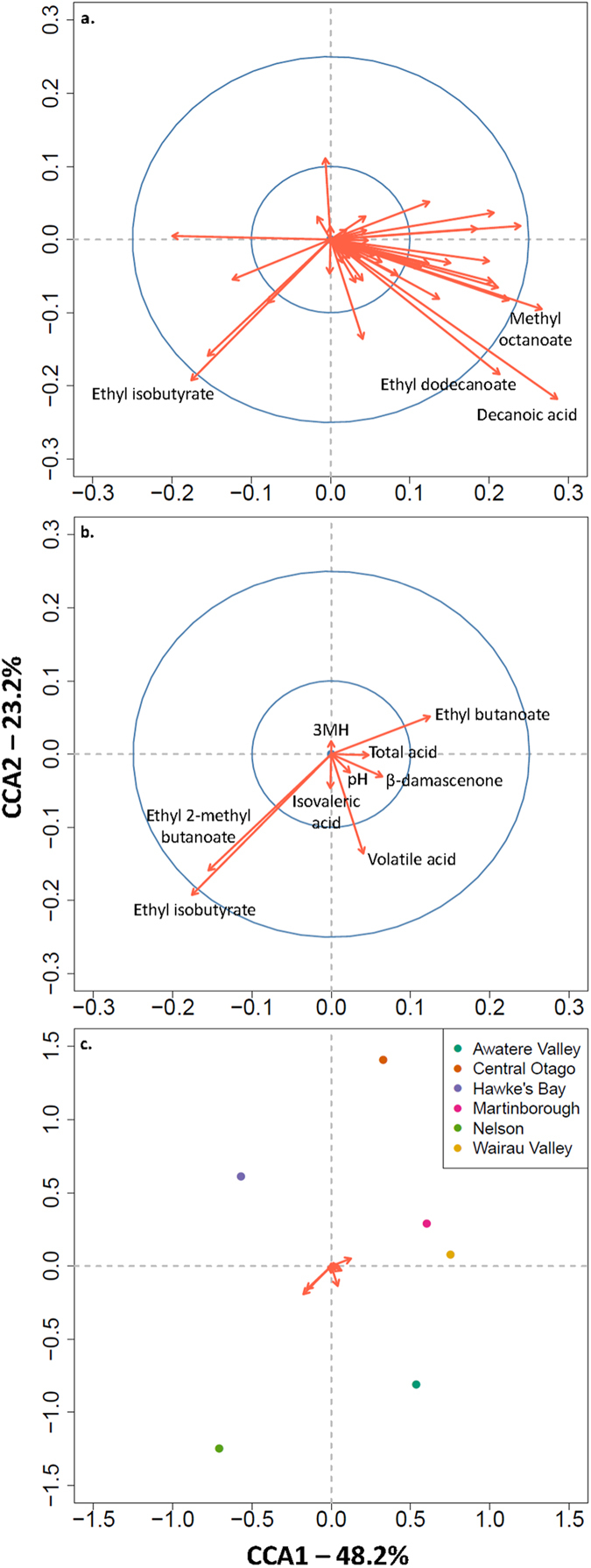
Visualization of the chemicals that individually explain more than 25% of the regional variation as calculated by ANOVA analyses. (**a**) The direction and magnitude of all chemical loading vectors, with labels for the chemicals that reported a magnitude above 0.25. The blue circles represent the position of 0.1 and 0.25. (**b**) The chemical loading vectors in the CCA plot for those that reported an R^2^ value for region larger than 0.25 and an *F*-statistics larger than 5 in the ANOVA analyses (Supplementary Table S4). The blue circles represent the position of 0.1 and 0.25. (**c**) The same chemical loading vectors reported in *b* with respect to the regional centers of the chemical profiles.

CCA additionally provides vectors indicating the direction and magnitude of influence that each chemical property has on the positioning of the sample aroma profiles within the plot, and potentially provides a mechanism to infer which chemicals differentiate each region. Four compounds (three esters and one fatty acid) have the greatest impact on the distribution of these wine phenotypes generally with vectors of a magnitude larger than 0.25 (Fig. 3a); however these chemical compounds are not necessarily correlated to the differentiation calculated between regions. To focus on and visualize the vectors of the chemical properties most important to the differences in chemical profiles between ferments conducted by yeasts derived from different regions we identified those chemicals that reported R^2^ values above 0.25, and *F*-statistics above 5 in the individual ANOVA analyses (Fig. 3b,c; Supplementary Table S4). This reveals that concentrations of ethyl isobutyrate and ethyl-2-methyl butanoate, which have apple and sweet fruit sensory descriptors, are on average both greatest in the ferments conducted by the genotypes deriving from Nelson and least in those from Central Otago and Martinborough. In addition, concentrations of ethyl butanoate (sensory descriptors of peach, apple and sweet) are on average greatest in ferments conducted by genotypes derived from Martinborough, and least in ferments conducted by genotypes derived from Nelson (Fig. 3). β-damascenone (sensory descriptors of apple, honey and floral) concentrations are on average greater in the ferments conducted by yeast genotypes derived from the Awatere and Wairau Valleys comprising the larger Marlborough region, and least from the ferments conducted by genotypes deriving from the Hawke’s Bay. Together this paints an intuitively sensible picture and reveals that the differential wine phenotype signatures driven by yeasts derived from different regions are not one-dimensional but multi-faceted.

### The genetic basis for differences in chemical profiles

While not exclusively genetically determined, the types and concentrations of metabolites produced by *S. cerevisiae* are significantly influenced by yeast genotype^37^,^38^,^39^. It is thus not surprising that a Mantel test evaluating the correlation between *S. cerevisiae* genotype genetic distance (using microsatellite profiles)^17^ and volatile chemical profile distance (calculated using Jaccard dissimilarity) reveal they are significantly correlated (R^2^ = 0.189; *P *< 0.0001). This formally allows us to accept the alternate hypothesis at the core of this study: that there is a significant correlation between the genetic relatedness of natural *S. cerevisiae* sub-populations and their effect on resulting wine phenotypes. Additionally, PERMANOVA analysis using the assignment of genotypes to inferred genetic clusters calculated using InStruct^17^ as a factor, as opposed to region of origin, increased the R^2^ value by 0.051 to 0.151 or 15% (*P* = 0.007). Some of the genotypes do not have a high proportion of ancestry to any one inferred population, and thus have mixed ancestry to different regions (Dataset S1). If these hybrid genotypes are removed and only those genotypes with a ‘clean’ geographic signal are analyzed, the PERMANOVA analysis reveals an increase in the R^2^ for the factor “region” to 0.198 (*P* = 0.006), double that of the original analysis (Table 1c).

### The effect of regionally co-fermented genotypes and blended wines on volatile profiles

There is evidence to show that the presence of other yeasts during fermentation, be they conspecifics or other species, may affect the subsequent volatile profiles of wine compared to the profiles produced when genotypes ferment in isolation^25^,^40^,^41^,^42^. We moved to evaluate whether interactions between genotypes from each region may affect and potentially alter regional signals for wine phenotypes. We compared the volatile profile of regional co-ferments, produced by inoculating all six genotypes from a region together in equal proportions, to regional blends, created by mixing the final wine produced by single genotypes from each region in equal proportions. PERMANOVA reveals that the type of ferment (co-ferment or blend) has a significant effect on chemical profiles (R^2^ = 0.061, *P* = 0.014; Table 1d). Again CCA was used to visualize the differences between the chemical profiles, and while overlap between the blends and co-ferments is evident, the blended ferments show less variability than the co-ferments, and are typically placed in the lower right of the plot (Supplementary Fig. S1a). Individual chemical ANOVA and the resulting CCA plot show the main differences between the co-ferments and blends are driven by ethyl decanoate, ethyl dodecanoate, ethyl octanoate and ethyl acetate (Supplementary Fig. S1b).

While the co-fermentation of multiple genotypes significantly affects the phenotype of wine compared to blending, it appears to erode signal for wine phenotype regionality, as PERMANOVA analysis reveals no strong regional co-ferment effect on volatile profiles (R^2^ = 0.346, *P* = 0.073; Table 1e). However, this may be an issue of statistical power—only three replicates of regional co-ferments and blends were implemented compared to the six volatile profiles from each of six genotypes from each region in the initial analysis. It is worth noting that the *P*-value for the effect of region reported by the co-ferments is marginal (*P* = 0.073), but the value for blends is not (*P* = 0.196) (Table 1 e,f), and might suggest that blending more greatly erodes any signal for regional wine phenotype than co-fermentation does.

## Discussion

We experimentally tested and quantified the extent to which genetically distinct regional populations of *S. cerevisiae* affect wine phenotype in terms of volatile composition. We show significant positive correlations between the genetic and geographic relatedness of natural *S. cerevisiae* sub-populations and their effect on resulting wine phenotypes. As far as we are aware this is the first empirical test for whether there is potential for a microbial aspect to *terroir*. This result aligns with the belief that microbes significantly contribute to the regional identity or *terroir* of wine and may potentially extend to the differential effects of microbes on other important agricultural crops and produce generally.

The ability of microbes to affect differential crop phenotypes is potentially greater than we estimate here. First, we have not evaluated microbes’ effect on crop development and how this might vary between differential geographic communities and populations. This is apparent in some sense, as different crops tend to suffer different levels of disease in different geographic areas; however the subtler effects of microbes on crop development and quality are mostly not understood. Moreover, many other species of fungi and bacteria contribute to the natural conversion of juice to wine and many of these also significantly affect wine phenotype, and there is good evidence to show these may synergistically interact^25^,^40^. Thus, the presence of regionally differentiated communities of yeast and bacteria associated with ripe fruit, as has been demonstrated^1^,^15^,^16^,^17^, may further affect differences in wine phenotype over that we have revealed here, but this remains to be evaluated. Here we conservatively remove both these effects as we use the same homogenized batch of grape juice and examine the ability of differential populations of just one species to manipulate crop produce. Even so, we provide evidence that different natural sub-populations of *S. cerevisiae* deriving from different regions have the potential to significantly and differentially affect wine phenotype.

The chemicals responsible for the differences between regions are not consistently from any particular class (Supplementary Table S4), and thus the microbially driven signals for difference in wine phenotype by region are complex, which makes intuitive sense. We attempted to evaluate the impact of how humans might perceive these differences in wine phenotypes by standardizing chemical concentrations with published OAVs^32^,^33^. This analysis again reported a significant effect of regionally differentiated microbes on wine phenotypes; however, OAVs are subjective to an extent, and interactions between chemicals that may lead to enhancement or masking of aromas are not accounted for here^33^. Ultimately the inclusion of sensory trials in these kinds of studies would add an extra layer to evaluate the extent that microbes play in the geographic differentiation of wine phenotypes. In addition this study only employs microbes that were determined to differ by region from just one year: how such population differentiation, and their resulting effects on crop phenotypes, changes across multiple years remains to be tested.

Recently a handful of studies have shown that the communities and populations of microbes associated with vines and wines vary by region^1^,^15^,^16^,^17^, and these are the first demonstrations of geographic variance in microbes associated with agriculture generally. Here we conduct a crucial follow-on to these observations: to test whether the genetic variance in microbial populations correlates with altered crop phenotypes. Geographic variance in crop physical and sensorial signatures are well described, and have important economic and consumer preference consequences^10^, but the drivers behind these differences have not been objectively evaluated and quantified. While we are not able to make any assertions regarding the temporal stability of these results, these data show there is a quantifiable microbial aspect to *terroir,* thus revealing the potential importance of microbial populations on the regional identity of wine, and may also extend to other important agricultural commodities. With a better understanding of the forces driving microbial population and community differentiation, food and agricultural sectors can develop systems to better control and manage these communities to help conserve the regional identity of products. More generally this finding indicates the importance of characterizing and understanding biodiversity and the services it may provide. Together this suggests that the long-term implementation of methods that maintain biodiversity may have tangible economic imperatives as well as being driven by a desire to employ agricultural practices that increase responsible environmental stewardship.

## Methods

### Genotype selection

Six genotypes were selected from six major wine growing regions in NZ to represent the genetic diversity in each region (See Fig. 1 for geographic locations). Here we specifically employed genotypes previously isolated from spontaneous ferments^17^. We used Bayesian analyses to select one genotype from each region that harbored at least one allele that was unique to that region while the remaining genotypes were selected to cover the diversity of ancestry profiles reported in each regional population as reported in Knight and Goddard (2015)^17^.

### Micro-fermentation

The 126 ferments were conducted in three batches due to space constraints, and each batch contained one replicate of every treatment. Each ferment contained 230 mL of Marlborough (NZ) Sauvignon Blanc juice from the 2012 vintage (pH = 3.1, 22.1 °Brix) sterilized with 200 μL/L Dimethyl dicarbonate (DMDC) and with the SO_2_ level adjusted to 10 mg/L. Each *S. cerevisiae* genotype was grown up independently in liquid YPD (1% yeast extract, 2% peptone, 2 % glucose) prior to inoculation. The live cell concentration of each culture was determined using a haemocytometer with methylene blue staining, and cells were inoculated to give a final concentration of 2.5 × 10^6^ cells/mL. Regional co-ferments were performed by inoculating all six genotypes isolated from each region in equal proportions to the same final concentration of 2.5 × 10^6^ cells/mL. Triplicate un-inoculated controls were included in each batch to control for weight loss via evaporation and to identify potential contamination issues. This totaled 126 experimental ferments and 9 un-inoculated controls. Ferments were conducted at 15 °C with 150 rpm shaking in 250 mL Erlenmeyer flasks with air-locks. Fermentation progress was monitored by weighing the flasks daily^30^ and ferments were considered finished when the rate of weight loss was below 0.001 g/hr (after controlling for evaporation as calculated from the controls) or when they reached 30 days. Ferments were centrifuged at 6000 × *g* for 10 minutes to pellet cells and the supernatant was decanted and frozen at −20 °C until chemical analyses were performed.

### Blends

After fermentation, regional blends were constructed from the single genotype ferments. Equal proportions of wine from ferments of each of the six genotypes from each region were homogenized, creating triplicate regional blends for each of the six regions. This resulted in a total of 144 wine samples for chemical analyses.

### Chemical analyses

Final ethanol concentration, pH, residual sugar, volatile acidity (VA) and titratable acidity (TA) were quantified using FTIR (Fourier Transform Infrared Spectroscopy) with a FOSS WineScan^TM^ FT120. The varietal thiols 3MH, 3MHA and 4MMP were quantified using an ethyl propiolate derivatization and analyzed on an Agilent 6890N gas chromatograph (Santa Clara, CA, USA) equipped with a 7683B automatic liquid sampler, a G2614A autosampler and a 593 mass selective detector as outlined in Herbst-Johnstone *et al.* (2013)^43^. Thirty-two esters, higher alcohols, terpenes, C6-alcohols and fatty acids were quantified simultaneously using a HS-SPME/GC-MS method outlined in Herbst-Johnstone *et al.* (2013a)^44^. Raw data was transformed with GCMSD Translator and peak integration was performed using MS Quantitative Analysis, both part of the Agilent MassHunter Workstation Software (Version B.04.00, Agilent Technologies).

### Statistical Analyses

The sigmoid or altered Gompertz decay function described by Tronchoni *et al.* (2009)^45^ was used to build a model of fermentation kinetics for each ferment from the weight loss data to infer the lag phase. The data was fitted using the non-linear least squares method implemented in the R package *nlstools*^46^. Differences in the lag phase between batches were tested using a mixed linear model in JMP (Version 10) accounting for genotype and stuck ferments as random factors.

Statistical tests for regional signal were performed on the chemical profiles for all datasets separately using a PERMANOVA approach as implemented in the R package *vegan*^47^. Jaccard distances were used to calculate pairwise distances in the model and 10 000 permutations of the raw data constrained at the genotype level to account for the dependency between genotypes and their replicates, were performed for the hypothesis tests (*F*- tests). Full factorial models were implemented and subsequently reduced upon analysis of the results to obtain the model of best fit. Pairwise PERMANOVA analyses were performed between all combinations of regions for the single genotype ferments. Since *P*-values can be misleading when multiple comparisons are performed^34^ we follow the idea that more emphasis should be placed on the magnitude of effects^35^ thus the *F*-statistics from these comparisons are used as a measure of the strength of evidence for a regional effect. Constrained Correspondence Analysis (CCA), implemented in the R package *vegan*, was used to visualize the data. This is analogous to a Principle Component Analysis in that transformations of the data are performed to provide components that allow the data to be visualized in 2-D plots. The CCA additionally partitions these components into a part that is explained by the specified linear model (in this case “region + batch”) and a part that is residual to that model. The plot that is produced rotates the data to the best orientation to observe the variation explained by the model. This method allows the PERMANOVA model to be built into the visualization, providing the most appropriate transformation and orientation of the data to visualize differences between the factors of interest.

A Mantel test was performed in GenAlEx (Genetic Analyses in Excel) version 6.5^48^,^49^ between a chemical distance matrix calculated using the Jaccard similarity coefficient, and the genetic distance matrix calculated using data from Knight and Goddard (2015)^17^.

## Additional Information

**How to cite this article**: Knight, S. *et al.* Regional microbial signatures positively correlate with differential wine phenotypes: evidence for a microbial aspect to *terroir*. *Sci. Rep.* **5**, 14233; doi: 10.1038/srep14233 (2015).

## Supplementary Material

Supplementary Information

Supplementary Table S1

Supplementary Table S2

Supplementary Dataset 1

## Figures and Tables

**Table 1:** Summary of all PERMANOVA analyses.

**Factors**	**Df**	***F* Model**	**R^2^**	***P*-value**
*(a) Single strain ferments only*				
** Region**	**5**	**2.056**	**0.100**	0.001***
** **Batch	2	3.687	0.072	0.001***
** **Region*Batch	9	0.860	0.076	0.093
** **Residuals	77		0.752	
** **Total	93		1	
*(b) Single strain ferments only, with chemicals standardised by OAV*				
** Region**	**5**	**2.758**	**0.127**	0.002**
** **Batch	2	4.302	0.079	0.001***
** **Region*Batch	9	0.987	0.082	0.166
** **Residuals	77		0.711	
** **Total	93		1	
*(c) Single strain ferments with strains with mixed ancestry removed*				
** Region**	**5**	**3.176**	**0.198**	0.006**
** **Batch	2	3.092	0.077	0.005**
** **Region*Batch	9	1.482	0.166	0.056
** **Residuals	45		0.560	
** **Total	61		1	
*(d) Co-ferments and blends only, testing for effect of the type of ferment*				
** Type**	**1**	**2.425**	**0.061**	0.014*
** **Batch	2	3.698	0.186	0.014*
** **Residuals	30		0.753	
** **Total	33		1	
*(e) All co-ferment samples only*				
** Region**	**5**	**1.555**	**0.346**	**0.073**
** **Batch	2	2.364	0.210	0.073
** **Residuals	10		0.444	
** **Total	17		1	
*(f) All blend samples only*				
** Region**	**5**	**1.375**	**0.339**	**0.196**
** **Batch	2	1.704	0.168	0.196
** **Residuals	10		0.493	
** **Total	17		1	

## References

[b1] Bokulich N. A., Thorngate J. H., Richardson P. M. & Mills D. A. Microbial biogeography of wine grapes is conditioned by cultivar, vintage, and climate. Proc Natl Acad Sci USA 111, E139–E148 (2014). 24277822 10.1073/pnas.1317377110 PMC3890796

[b2] López-Rituerto E. *et al.* Investigations of La Rioja *terroir* for wine production using 1H NMR metabolomics. J Agric Food Chem 60, 3452–3461 (2012). 22397579 10.1021/jf204361d

[b3] Robinson A. L. *et al.* Influence of geographic origin on the sensory characteristics and wine composition of *Vitis vinifera* cv. Cabernet Sauvignon wines from Australia. Am. J. Enol. Vitic. 63, 467–476 (2012).

[b4] Son H.-S. *et al.* Metabolomic studies on geographical grapes and their wines using 1H NMR analysis coupled with multivariate statistics. J Agric Food Chem 57, 1481–1490 (2009). 19192969 10.1021/jf803388w

[b5] Benkwitz F. *et al.* Identifying the chemical composition related to the distinct aroma characteristics of New Zealand Sauvignon blanc wines. Am. J. Enol. Vitic. 63, 62–72 (2012).

[b6] Lund C. M. *et al.* New Zealand Sauvignon blanc distinct flavor characteristics: Sensory, chemical, and consumer aspects. Am. J. Enol. Vitic. 60, 1–12 (2009).

[b7] Obuchowicz J., Engelhardt U. H. & Donnelly K. Flavanol database for green and black teas utilising ISO 14502-1 and ISO 14502-2 as analytical tools. J Food Compost Anal 24, 411–417 (2011).

[b8] Torres-Moreno M., Torrescasana E., Salas-Salvadó J. & Blanch C. Nutritional composition and fatty acids profile in cocoa beans and chocolates with different geographical origin and processing conditions. Food Chem 166, 125–132 (2015). 25053037 10.1016/j.foodchem.2014.05.141

[b9] Costa Freitas A. M. & Mosca A. I. Coffee geographic origin — an aid to coffee differentiation. Food Res Int 32, 565–573 (1999).

[b10] Van Leeuwen C. & Seguin G. The concept of Terroir in viticulture. J Wine Res 17, 1–10 (2006).

[b11] Philippot L., Raaijmakers J. M., Lemanceau P. & van der Putten W. H. Going back to the roots: the microbial ecology of the rhizosphere. Nat Rev Microbiol 11, 789–799 (2013). 24056930 10.1038/nrmicro3109

[b12] Peiffer J. A. *et al.* Diversity and heritability of the maize rhizosphere microbiome under field conditions. Proc Natl Acad Sci USA 110, 6548–6553 (2013). 23576752 10.1073/pnas.1302837110 PMC3631645

[b13] Whipps J. M. Microbial interactions and biocontrol in the rhizosphere. J Exp Bot 52, 487–511 (2001). 11326055 10.1093/jexbot/52.suppl_1.487

[b14] Fleet G. H. in The yeast handbook volume 2: Yeasts in food and beverages (eds Querol A. & Fleet G. H. ) 1–12 (Springer-Verlag: Berlin Heidelberg, , 2006).

[b15] Gayevskiy V. & Goddard M. R. Geographic delineations of yeast communities and populations associated with vines and wines in New Zealand. ISME J 6, 1281–1290 (2012). 22189497 10.1038/ismej.2011.195 PMC3379632

[b16] Taylor M. W., Tsai P., Anfang N., Ross H. A. & Goddard M. R. Pyrosequencing reveals regional differences in fruit-associated fungal communities. Environ Microbiol 16, 2848–2858 (2014). 24650123 10.1111/1462-2920.12456 PMC4257574

[b17] Knight S. & Goddard M. R. Quantifying separation and similarity in a *Saccharomyces cerevisiae* metapopulation. ISME J 9, 361–370 (2015). 25062126 10.1038/ismej.2014.132 PMC4303630

[b18] Barata A., Malfeito-Ferreira M. & Loureiro V. The microbial ecology of wine grape berries. Int J Food Microbiol 153, 243–259 (2012). 22189021 10.1016/j.ijfoodmicro.2011.11.025

[b19] Dubourdieu D., Tominaga T., Masneuf I., Des Gachons C. P. & Murat M. L. The role of yeasts in grape flavor development during fermentation: The example of Sauvignon blanc. Am. J. Enol. Vitic. 57, 81–88 (2006).

[b20] Swiegers J. H. *et al.* The influence of yeast on the aroma of Sauvignon Blanc wine. Food Microbiol 26, 204–211 (2009). 19171264 10.1016/j.fm.2008.08.004

[b21] Lambrechts M. G. & Pretorius I. S. Yeast and its importance to wine aroma - a review. South African Journal of Enology and Viticulture 21, 97–129 (2000).

[b22] Swiegers J. H., Bartowsky E. J., Henschke P. A. & Pretorius I. S. Yeast and bacterial modulation of wine aroma and flavour. Aust. J. Grape Wine Res. 11, 139–173 (2005a).

[b23] Sumby K. M., Grbin P. R. & Jiranek V. Microbial modulation of aromatic esters in wine: Current knowledge and future prospects. Food Chem 121, 1–16 (2010).

[b24] Howell K. S. *et al.* Variation in 4-mercapto-4-methyl-pentan-2-one release by *Saccharomyces cerevisiae* commercial wine strains. FEMS Microbiol Lett 240, 125–129 (2004). 15522498 10.1016/j.femsle.2004.09.022

[b25] Anfang N., Brajkovich M. & Goddard M. R. Co-fermentation with *Pichia kluyveri* increase varietal thiol concentrations in Sauvignon Blanc. Aust. J. Grape Wine Res. 15, 1–8 (2009).

[b26] Goddard M. R. Quantifying the complexities of *Saccharomyces cerevisiae*'s ecosystem engineering via fermentation. Ecology 89, 2077–2082 (2008). 18724717 10.1890/07-2060.1

[b27] Pretorius I. S. Tailoring wine yeast for the new millennium: novel approaches to the ancient art of winemaking. Yeast 16, 675–729 (2000). 10861899 10.1002/1097-0061(20000615)16:8<675::AID-YEA585>3.0.CO;2-B

[b28] Goddard M. R., Anfang N., Tang R., Gardner R. C. & Jun C. A distinct population of *Saccharomyces cerevisiae* in New Zealand: evidence for local dispersal by insects and human-aided global dispersal in oak barrels. Environ Microbiol 12, 63–73 (2010). 19691498 10.1111/j.1462-2920.2009.02035.x

[b29] Gayevskiy V., Klaere S., Knight S. & Goddard M. R. ObStruct: A method to objectively analyse factors driving population structure using Bayesian ancestry profiles. PLoS One 9, e85196 (2014). 24416362 10.1371/journal.pone.0085196 PMC3887034

[b30] El Haloui N., Picque D. & Corrieu G. Alcoholic fermentation in winemaking: On-line measurement of density and carbon dioxide evolution. J Food Eng 8, 17–30 (1988).

[b31] Malherbe S., Watts V., Nieuwoudt H. H., Bauer F. F. & Toit M. D. U. Analysis of volatile profiles of fermenting grape must by headspace solid-phase dynamic extraction coupled with gas chromatography-mass spectrometry (HS-SPDE GC-MS): Novel application to investigate problem fermentations. J Agric Food Chem 57, 5161–5166 (2009). 19469561 10.1021/jf900532v

[b32] Swiegers J. H. & Pretorius I. S. in Advances in Applied Microbiology Vol. 57 (eds Bennett Joan W., Laskin Allen I. & Gadd Geoffrey M. ) 131–175 (Academic Press, 2005). 16002012 10.1016/S0065-2164(05)57005-9

[b33] Francis I. L. & Newton J. L. Determining wine aroma from compositional data. Aust. J. Grape Wine Res. 11, 114–126 (2005).

[b34] Krzywinski M. & Altman N. Points of significance: Comparing samples - part II. Nat Methods 11, 355–356 (2014). 10.1038/nmeth.2858 24724163

[b35] Nuzzo R. Scientific method: statistical errors. Nature 506, 150–152 (2014). 24522584 10.1038/506150a

[b36] Ter Braak C. J. F. Canonical correspondence analysis: A new eigenvector technique for multivariate direct gadient analysis. Ecology 67, 1167–1179 (1986).

[b37] Richter C. L., Dunn B., Sherlock G. & Pugh T. Comparative metabolic footprinting of a large number of commercial wine yeast strains in Chardonnay fermentations. FEMS Yeast Res 13, 394–410 (2013). 23528123 10.1111/1567-1364.12046

[b38] Pretorius I. S., Curtin C. D. & Chambers P. J. The winemaker’s bug. The winemaker’s bug 3, 149–158 (2012). 10.4161/bbug.19687 PMC3370933 22572786

[b39] Camarasa C., Sanchez I., Brial P., Bigey F. & Dequin S. Phenotypic landscape of Saccharomyces cerevisiae during wine fermentation: Evidence for origin-dependent metabolic traits. PLoS ONE 6 (2011). 10.1371/journal.pone.0025147 PMC3174997 21949874

[b40] Howell K. S., Cozzolino D., Bartowsky E. J., Fleet G. H. & Henschke P. A. Metabolic profiling as a tool for revealing *Saccharomyces* interactions during wine fermentation. FEMS Yeast Res. 6, 91–101 (2006). 16423074 10.1111/j.1567-1364.2005.00010.x

[b41] Cheraiti N., Guezenec S. & Salmon J. M. Redox interactions between Saccharomyces cerevisiae and Saccharomyces uvarum in mixed culture under enological conditions. Appl Environ Microbiol 71, 255–260 (2005). 15640195 10.1128/AEM.71.1.255-260.2005 PMC544210

[b42] Barrajón N., Capece A., Arévalo-Villena M., Briones A. & Romano P. Co-inoculation of different Saccharomyces cerevisiae strains and influence on volatile composition of wines. Food Microbiol 28, 1080–1086 (2011). 21569955 10.1016/j.fm.2011.02.016

[b43] Herbst-Johnstone M., Piano F., Duhamel N., Barker D. & Fedrizzi B. Ethyl propiolate derivatisation for the analysis of varietal thiols in wine. J Chromatogr A 1312, 104–110 (2013). 24034138 10.1016/j.chroma.2013.08.066

[b44] Herbst-Johnstone M. *et al.* Effects of mechanical harvesting on 'Sauvignon blanc' aroma. Acta Hort 978, 179–186 (2013a).

[b45] Tronchoni J., Gamero A., Arroyo-López F. N., Barrio E. & Querol A. Differences in the glucose and fructose consumption profiles in diverse Saccharomyces wine species and their hybrids during grape juice fermentation. Int J Food Microbiol 134, 237–243 (2009). 19632733 10.1016/j.ijfoodmicro.2009.07.004

[b46] Bates D. M. & Watts D. G. Nonlinear regression analysis and its applications. (Wiley, 1988).

[b47] Anderson M. J. A new method for non-parametric multivariate analysis of variance. Austral Ecol 26, 32–46 (2001).

[b48] Peakall R. & Smouse P. GenAlEx 6.5: Genetic analysis in Excel. Population genetic software for teaching and research – an update. Bioinformatics 28, 2537–2539 (2012). 22820204 10.1093/bioinformatics/bts460 PMC3463245

[b49] Peakall R. & Smouse P. E. GENALEX 6: Genetic analysis in Excel. Population genetic software for teaching and research. Mol Ecol Notes 6, 288–295 (2006). 10.1093/bioinformatics/bts460 PMC3463245 22820204

