# Supplementary material for: Regional microbial signatures positively correlate with differential wine phenotypes: evidence for a microbial aspect to terroir
Source: Sci Rep. 2015 Sep 24;5:14233. doi: 10.1038/srep14233 (PMC4585847; doi:10.1038/srep14233)
Supplement: Supplementary Information [file srep14233-s1.pdf]

Supplementary information

Manuscript title: Regional microbial signatures positively correlate with differential wine phenotypes: evidence for a microbial aspect to *terroir*

Sarah Knight, Steffen Klaere, Bruno Fedrizzi and Matthew R Goddard

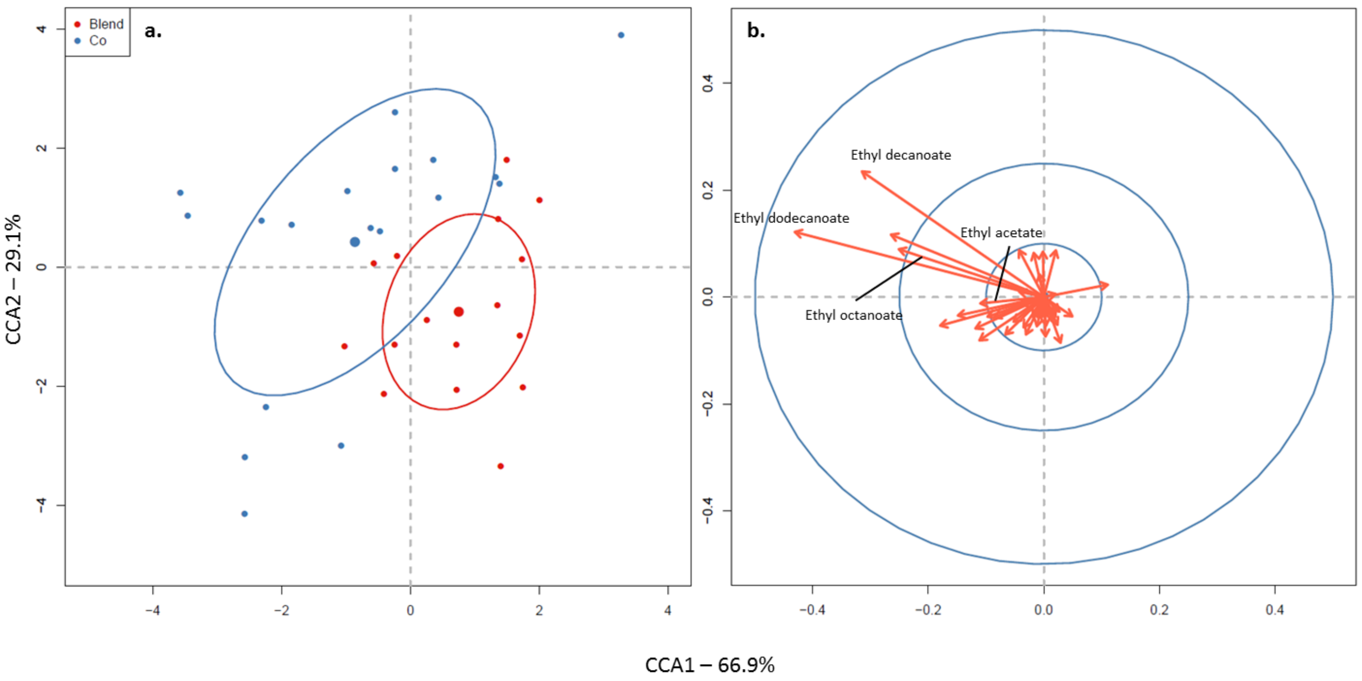

Supplementary Figure S1 – The CCA plot visualizing the differences between blends and co-ferments. (a) All sample points and 50 % ellipses for blends (red) and co-ferments (blue). (b) The direction and magnitude of all chemical loading vectors with labels provided for those that reported an R^2 value above 0.25 and an *F*-statistic above 5 for the factor “type” in ANOVA analyses. Ethyl acetate is the only chemical with a R^2 value above 0.25 that does not have a loading vector in the CCA plot above 0.25 in magnitude.

Supplementary Table S1: The Bayesian ancestry profiles resulting from InStruct analyses in Knight and Goddard (2015). The geographic regions largely represented by each inferred population are indicated above the column headings with the color referring to the colors seen in Figure 1 of Knight and Goddard (2015). The genotypes tested in this study are highlighted. The numbers refer to the proportion of ancestry for each genotype that is attributed to each inferred population.

- This table is provided as a separate Excel spreadsheet

Supplementary Table S2: The frequency of alleles for each locus by region of all individuals tested in Knight and Goddard (2015). Highlighted alleles are represented by the tested genotypes in this analysis. The proportion of each population that the selected genotypes cover in terms of the alleles present is calculated at the base of each table. The column headings refer to abbreviated region names corresponding to Awa = Awatere Valley, CO = Central Otago, HB = Hawke's Bay, Mart = Martinborough, Nel = Nelson, Wai = Wairau Valley.

- This table is provided as a separate Excel spreadsheet

Supplementary Table S3 – Regional pairwise PERMANOVA results showing the R^2 value and *F*-statistic for the factor "region".

**Region 1**	**Region 2**	**R^2**	***F* Model**
Awatere Valley	Central Otago	0.067	1.64
Awatere Valley	Hawke's Bay	0.057	1.77
Awatere Valley	Martinborough	0.030	0.91
Awatere Valley	Nelson	0.032	1.00
Awatere Valley	Wairau Valley	0.026	0.64
Central Otago	Hawke's Bay	0.063	2.33
Central Otago	Martinborough	0.076	2.97
Central Otago	Nelson	0.121	5.03
Central Otago	Wairau Valley	0.063	1.83
Hawke's Bay	Martinborough	0.072	3.21
Hawke's Bay	Nelson	0.077	3.47
Hawke's Bay	Wairau Valley	0.037	1.28
Martinborough	Nelson	0.078	3.60
Martinborough	Wairau Valley	0.056	2.04
Nelson	Wairau Valley	0.077	2.88

Supplementary Table S4 – Results from the ANOVA analyses of the single genotype ferments for each individual chemical. Each chemical was tested using the full factorial model of the factors "batch" and "region". *F*-statistics and R^2 values for both the factor "region" and the whole model are reported. Compounds highlighted in red report a R^2 value above 0.25 and an *F*-statistic above 5 and the vectors for these in the CCA analysis are visualized in Fig.3b and c.

Chemical	Class	Model R^2	Model $F_{25,68}$	Region R^2	Region $F_{5,68}$
Ethanol		0.582	6.70	0.167	6.15
**Total Acid**		**0.370**	**2.82**	**0.322**	**7.86**
**Volatile Acid**		**0.371**	**2.84**	**0.325**	**7.97**
**pH**		**0.402**	**3.24**	**0.380**	**9.80**
Residual Sugar		0.189	1.12	0.078	1.48
3MHA	Thiol	0.342	2.51	0.160	3.74
**3MH**	**Thiol**	**0.342**	**2.50**	**0.255**	**5.96**
**Ethyl isobutyrate**	**Ester**	**0.394**	**3.13**	**0.346**	**8.81**
**Ethyl butanoate**	**Ester**	**0.381**	**2.96**	**0.253**	**6.30**
**Ethyl 2-methyl butanoate**	**Ester**	**0.415**	**3.41**	**0.318**	**8.37**
Ethyl isovalerate	Ester	0.242	1.53	0.120	2.44
Ethyl hexanoate	Ester	0.390	3.08	0.114	2.88
Ethyl octanoate	Ester	0.332	2.39	0.137	3.16
Ethyl decanoate	Ester	0.248	1.59	0.064	1.31
Ethyl dodecanoate	Ester	0.153	0.87	0.032	0.58
Ethyl acetate	Ester	0.234	1.47	0.115	2.32
Isobutyl acetate	Ester	0.119	0.65	0.056	0.98
Isoamyl acetate	Ester	0.357	2.68	0.178	4.26
Hexyl acetate	Ester	0.522	5.25	0.175	5.65
Cis-3-hexenyl acetate	Ester	0.415	3.42	0.098	2.57
Ethyl phenylacetate	Ester	0.268	1.76	0.086	1.80
β-phenylethyl acetate	Ester	0.284	1.91	0.160	3.45
Methyl octanoate	Ester	0.317	2.24	0.146	3.30
Isobutanol	Alcohol	0.183	1.07	0.110	2.08
Isoamylalcohol	Alcohol	0.327	2.34	0.108	2.47
Methionol	Alcohol	0.384	2.99	0.083	2.06
Phenylethyl alcohol	Alcohol	0.152	0.86	0.112	2.04
**β-damascenone**	**Norisoprenoids/Terpene**	**0.381**	**2.96**	**0.307**	**7.62**
β-ionone	Norisoprenoids/Terpene	0.185	1.09	0.045	0.85
Cis/trans-rose-oxide	Norisoprenoids/Terpene	0.247	1.58	0.188	3.85
Linalool	Norisoprenoids/Terpene	0.194	1.16	0.057	1.09
β-citronellol	Norisoprenoids/Terpene	0.173	1.01	0.088	1.64
Hexanol	C6 Compound	0.156	0.89	0.095	1.73
Trans-3-hexen-1-ol	C6 Compound	0.216	1.32	0.118	2.31
Cis-3-hexen-1-ol	C6 Compound	0.254	1.64	0.063	1.29
**Isovaleric acid**	**Fatty Acid**	**0.431**	**3.64**	**0.371**	**10.03**
Hexanoic acid	Fatty Acid	0.282	1.89	0.175	3.75
Octanoic acid	Fatty Acid	0.273	1.81	0.180	3.81
Decanoic acid	Fatty Acid	0.255	1.64	0.104	2.15

Dataset S1: Chemical concentrations and factors used in the analysis

This additional excel spreadsheet contains the chemical concentrations for each sample and factors that were utilized in the analysis. Under the column “Stuck”: F = Finished, S = Stuck.
